# Circulating Ionized Magnesium: Comparisons with Circulating Total Magnesium and the Response to Magnesium Supplementation in a Randomized Controlled Trial

**DOI:** 10.3390/nu12010263

**Published:** 2020-01-20

**Authors:** Mary R. Rooney, Kyle D. Rudser, Alvaro Alonso, Lisa Harnack, Amy K. Saenger, Pamela L. Lutsey

**Affiliations:** 1 Division of Epidemiology & Community Health, University of Minnesota, Minneapolis, MN 55454, USA, harna001@umn.edu (L.H.); lutsey@umn.edu (P.L.L.); 2 Department of Epidemiology and Welch Center for Prevention, Epidemiology, & Clinical Research, Johns Hopkins University, Baltimore, MD 21287, USA; 3 Division of Biostatistics, School of Public Health, University of Minnesota, Minneapolis, MN 55455, USA, rudser@umn.edu; 4 Department of Epidemiology, Rollins School of Public Health, Emory University, Atlanta, GA 30322, USA, alvaro.alonso@emory.edu; 5 Department of Laboratory Medicine and Pathology, Hennepin Healthcare, Minneapolis, MN 55415, USA, amy.saenger@hcmed.org

**Keywords:** ionized magnesium, total magnesium, randomized controlled trial, magnesium supplement, nutritional epidemiology

## Abstract

Ionized Mg (iMg) is considered the biologically active fraction of circulating total Mg (tMg). It is possible that iMg may be a more physiologically relevant marker than tMg. Using data from a double-blind pilot randomized controlled trial, we tested (1) whether oral Mg supplementation will increase iMg concentrations compared with placebo and (2) the relationship between iMg and tMg at baseline. Additionally, we evaluated the agreement between iMg measured in fresh whole blood versus stored samples. A total of fifty-nine participants were randomized 1:1 to oral Mg supplementation (400 mg/day, Mg Oxide) or placebo for 10 weeks. Fasting blood samples were obtained at baseline and follow-up. The analysis used linear regression and an intent-to-treat approach. Participants were generally healthy, the mean age was 62, and 73% were female. The baseline iMg and tMg were modestly and positively associated (r = 0.50). The ratio of baseline iMg to tMg was 64%. The mean supplement effect on iMg was 0.03 mmol/L (95% CI:0.01, 0.05) for Mg supplementation versus placebo. The supplement effect on iMg was not statistically significantly different according to baseline iMg status (above/below median). Compared to fresh blood, iMg was consistently higher in refrigerated and frozen samples by 0.14 and 0.20 mmol/L, respectively. In this relatively healthy adult population, Mg supplementation over 10 weeks resulted in increased iMg concentrations. Whether iMg is a more appropriate measure of Mg status than tMg, and the public health or clinical utility of measuring iMg remains to be determined.

## 1. Introduction

Magnesium (Mg) homeostasis reflects a complex and dynamic interplay between dietary intake, absorption, and excretion [1,2]. The majority of total body Mg resides within the bone tissue, while less than 1% of total body Mg lies extra-cellularly. Serum total Mg (tMg) has traditionally been used to assess Mg status in both clinical and research settings, with a reference range of 0.75–0.95 mmol/L (multiply mmol/L by 2.43 for mg/dL; 1.82–2.31 mg/dL) [3]. There are important considerations to be cognizant of when using tMg to reflect Mg status. Of the circulating tMg in serum, approximately 20%–30% is bound to proteins and is thought to be physiologically inactive. Ionized Mg (iMg) constitutes approximately 60%–70% of circulating tMg [4,5] and is considered the biologically active form of circulating Mg [6]. It is possible that iMg may be a more physiologically relevant marker than tMg [4,5].

iMg is infrequently measured in research or clinical settings [3,4], likely because it requires an immediate analysis of whole blood iMg, specialized equipment is required for measurement, and iMg measurement can be prone to interference by individual-level factors, such as pH and serum calcium. While tMg and iMg generally correlate in normal, healthy subjects, the literature has been mixed in both observational studies and randomized controlled trials (RCTs) of Mg supplementation [7,8,9,10]. Furthermore, these studies have primarily been conducted in populations with comorbidities thought to influence Mg homeostasis.

Since relatively little is known about iMg in healthy populations, using data from a Mg supplementation RCT, we tested the following hypotheses: (1) oral Mg supplementation will increase iMg and tMg concentrations compared with placebo, particularly in those with low baseline iMg and tMg concentrations, respectively; and (2) iMg and tMg will be modestly associated at baseline and in response to supplementation. Additionally, to better understand considerations related to iMg laboratory analysis, we evaluated the agreement between iMg concentrations measured in fresh whole blood compared to refrigerated or frozen samples.

## 2. Materials and Methods

### 2.1. Study Design

We examined the interrelations between iMg and tMg, overall and in response to supplementation, using data from a pilot RCT entitled ‘Magnesium Supplementation for the Prevention of Supraventricular Arrhythmias’ [11]. This double-blind trial examined oral Mg supplementation for the primary prevention of supraventricular arrhythmias. The study was registered at Clinicaltrials.gov with the registration number NCT02837328. The study protocol was approved by the University of Minnesota Institutional Review Board (#1605M87323). All participants provided written informed consent.

A participant flow chart is shown in Supplemental Figure S1. Between March and June of 2017, 59 relatively healthy individuals from the general population aged >55 years and with no prior history of cardiovascular disease were randomized 1:1 to 400 mg/day of oral Mg (in the form of Mg oxide) or lactose placebo for 10 weeks. A block randomization scheme within two strata of age classification (<65 y and ≥65 y) was used. Within each stratum, randomly permuted block sizes of 2, 4, or 6 were used to generate the randomization schedule.

At the baseline visit, blood was drawn, weight, height, and blood pressure were measured, and several questionnaires were administered. The study treatment was mailed to participants 2 weeks after the baseline visit and the intervention then ensued. After 10 weeks on study treatment, participants returned for a second blood draw.

At the follow-up visit, participants brought the bottle containing the supplement or matching placebo, and treatment compliance was estimated by a pill count. Further details of the trial have been previously published [11], including measures of adverse effects and an assessment of blinding.

### 2.2. Biomarker Measures

Fasted (>8 h) blood samples were obtained at baseline and at the follow-up visit. The time of the blood draw was recorded. iMg was measured in whole blood approximately 10 min after collection using the pHOx^®^ Ultra blood gas analyzer. The pHOx^®^ Ultra blood gas analyzer provides results for the measured iMg concentration and the iMg concentration adjusted for pH (i.e., normalized iMg concentrations). As the concentration and activity of iMg can differ by sample pH, we present, herein, normalized iMg concentrations, except where indicated otherwise. For serum, blood specimens were allowed to clot and were then centrifuged, and serum aliquots were prepared. Serum tMg measurements were performed following the enrollment and completion of the study using a colorimetric assay on the Roche cobas c501 analyzer (Roche Diagnostics, Indianapolis, IN, USA) in the Advanced Research and Diagnostics Laboratory at the University of Minnesota. Ionized calcium (iCa) was also measured in whole blood using the pHOx^®^ Ultra blood gas analyzer and was adjusted for pH (i.e., normalized iCa concentrations).

To assess the impact of specimen stability on iMg concentrations, a sub-sample (n = 39) of split specimens were measured in serum samples, which had been refrigerated at 4 °C for approximately 1 h and stored in the freezer at −80 °C. Freezer specimens were measured ‘in batch’ at the end of the study (median 12 weeks after collection and storage). The time that specimens were placed in the refrigerator and freezer was recorded, as was the time of the iMg measurements. 

### 2.3. Statistical Analysis

Mean and median iMg concentrations at baseline are reported overall and by the treatment group. Baseline characteristics across study treatment arms and across baseline iMg concentrations above/below the median are also reported.

We used a linear regression model to test whether the change in iMg differs by treatment group. The change in iMg was the dependent variable with treatment group as an indicator variable adjusted for age stratum (randomization stratification factor, <65 y vs. ≥65 y) and baseline iMg concentration. Baseline iMg was included as a covariate for added precision [12,13]. Confidence intervals were based on robust variance estimation. Pre-specified subgroup analyses to assess whether the intervention effect differs by baseline iMg status (above/below median) were also conducted by including a cross-product term in the model (treatment group * baseline iMg status). Results for change in tMg are also provided, as previously reported [11], using this approach. Our primary analysis for change in iMg was based on the intent-to-treat principle. In secondary analyses, we excluded 21 participants who did not take at least 80% of the supplements as instructed. In post-hoc analyses, we additionally adjusted for sex and ionized calcium (separately).

To examine the baseline associations of iMg with tMg, we used a linear model with iMg as the dependent variable and tMg as the predictor variable with adjustment for treatment group, age stratum (<65 y vs. ≥65 y), and baseline iMg concentration. We used the slope to examine the association between iMg and tMg. Additionally, we used Pearson’s partial correlation coefficients, overall (adjusted for treatment arm, age, and sex) and by treatment group (adjusted for age and sex), for the baseline iMg and tMg. A scatter plot was used to visualize the association of baseline iMg and tMg. Additionally, Bland-Altman plots were used to visualize the comparative agreement of iMg in response to supplementation stratified by the treatment arm. A similar set of Bland-Altman plots were used for tMg.

To evaluate whether iMg concentrations differ according to sample processing method we report the mean/median concentrations for iMg measured from fresh whole blood after 1 h refrigeration and following one freeze–thaw cycle. We report, by processing method, the mean difference (95% confidence intervals) in iMg concentrations and the distribution of the difference in percentiles. We also report the mean time from blood draw to processing. We used Bland-Altman plots to visualize the agreement between iMg quantified in whole blood soon after blood draw with serum iMg, as measured from samples stored in the refrigerator and samples stored in the freezer.

Two-tailed *p*-values < 0.05 were considered statistically significant. STATA version 14.1 was used for analyses (College Station, TX, USA).

## 3. Results

### 3.1. Study Participants

Table 1 describes study participant characteristics at baseline by treatment group and by baseline iMg status (above and below the median). Aside from sex, baseline characteristics by treatment group were largely similar; the group randomized to Mg supplements was comprised of 86.2% women, while the group randomized to placebo was 60.0% women.

The average baseline iMg to tMg ratio was 64%. The median baseline iMg and tMg concentrations in the treatment group were 0.56 mmol/L (Percentile: 25th = 0.50 mmol/L, 75th = 0.60 mmol/L), and 0.86 mmol/L (25th = 0.82 mmol/L, 75th = 0.90 mmol/L), respectively. In the placebo group, baseline iMg was 0.54 mmol/L (25th = 0.52 mmol/L, 75th = 0.57 mmol/L) and tMg was 0.86 mmol/L (25th = 0.82 mmol/L, 75th = 0.90 mmol/L). Baseline characteristics stratified by baseline iMg status above or below the median were comparable.

### 3.2. Effect of Magnesium Supplementation on Magnesium Biomarkers

Table 2 shows the mean and standard deviations (SD) for iMg and tMg concentrations at baseline, follow-up, and the change in iMg and tMg by treatment arm. Also presented in Table 2 are age- and baseline-adjusted differences according to assigned treatment arm. At the end of the intervention period, the change in iMg for those randomized to 400 mg/day of supplemental Mg was significantly higher than the change for those randomized to placebo [mean supplement effect = 0.03 mmol/L (95% CI:0.01, 0.05); *p*-value = 0.009]. As context, this effect estimate of 0.03 mmol/L corresponds to ~60% of 1 SD (0.05 mmol/L) of baseline iMg in this study. The supplement effect on iMg did not statistically significantly differ by baseline iMg concentrations (Supplemental Table S1; above vs. below the median, p-interaction = 0.86). As previously reported [11], there was a significant supplement effect on tMg of 0.04 mmol/L (0.01, 0.06); *p*-value = 0.004; this corresponds to ~66% of 1 SD (0.06 mmol/L) of baseline tMg. The supplement effect on tMg did not differ significantly by baseline tMg status (Supplemental Table S1; p-interaction = 0.27).

In secondary analyses, among the 38 participants with compliance >80% (based on pill count), the results were largely similar (Supplemental Table S2). When we adjusted the intervention effect for sex and baseline ionized calcium, the results were also largely similar (data not shown).

### 3.3. Relationship between Magnesium Biomarkers

**Figure 1:**
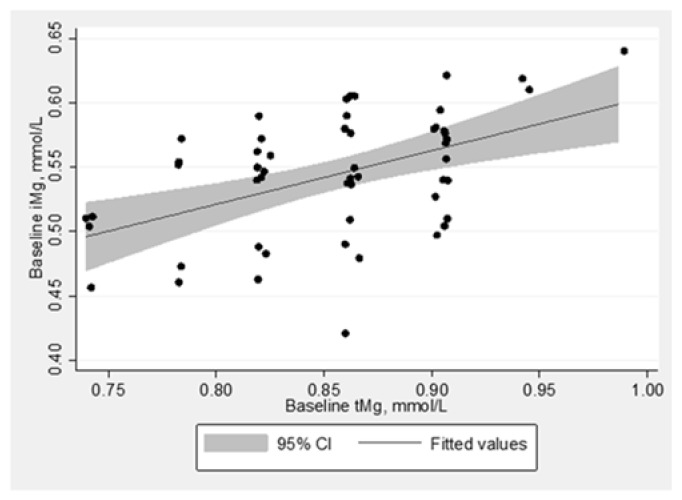
Scatterplot and linear fitted line between ionized and total magnesium at baseline, unadjusted, n = 49. Normalized ionized Mg (iMg) concentration, which is adjusted for blood pH.

**Figure 2:**
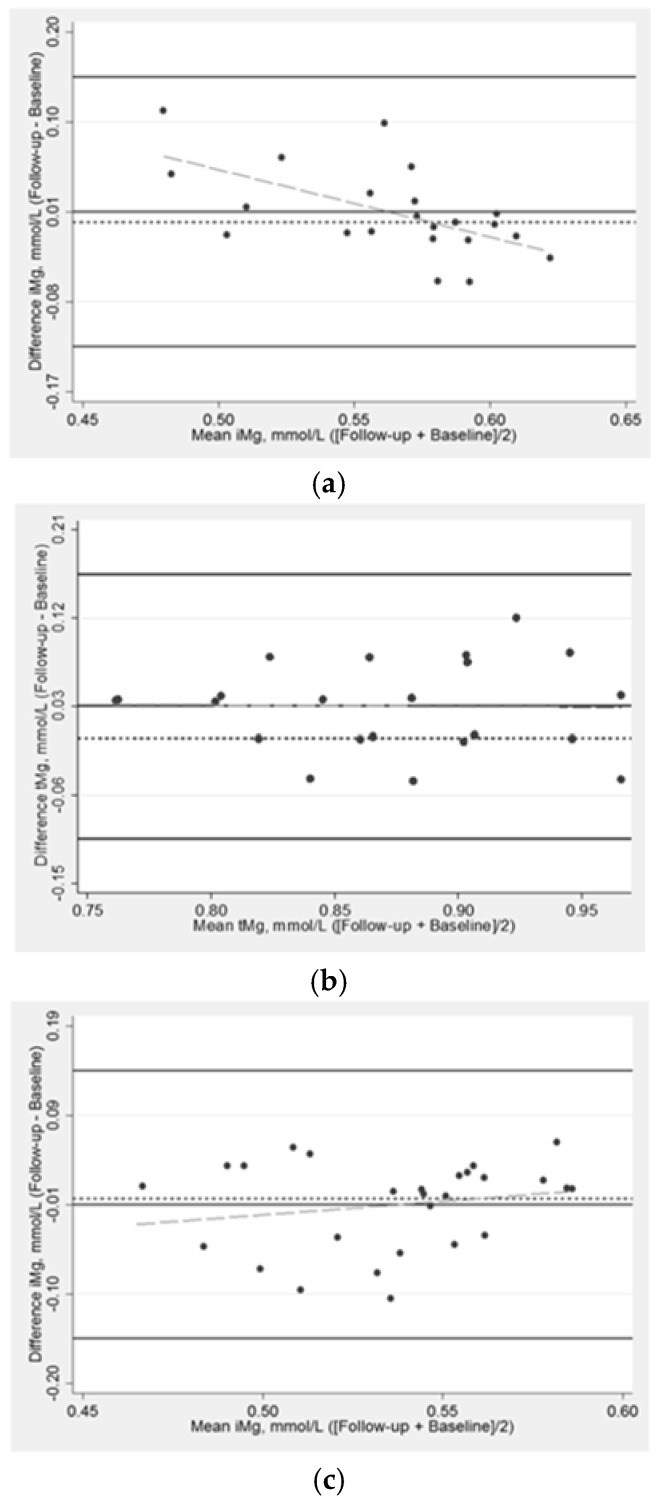

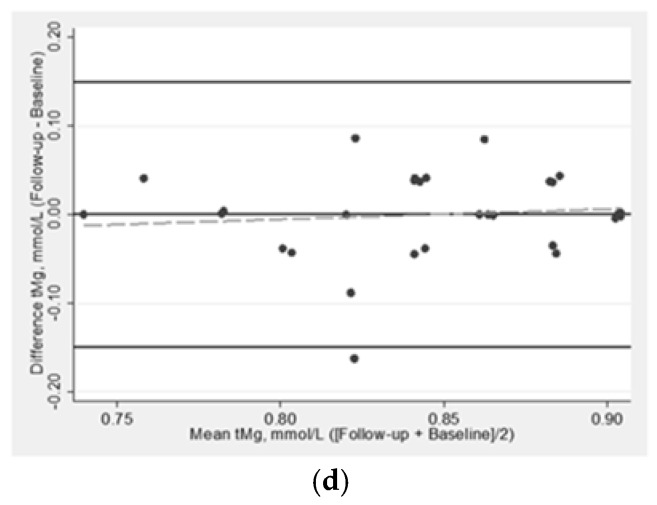
Bland-Altman plot assessing the (**a**) change in ionized magnesium in response to magnesium supplementation over 10 weeks in the treatment arm, *n* = 22; (**b**) change in total magnesium in response to magnesium supplementation over 10 weeks in the treatment arm, *n* = 24; (**c**) change in ionized magnesium in response to magnesium supplementation over 10 weeks in the placebo arm, *n* = 27; (**d**) change in total magnesium in response to magnesium supplementation over 10 weeks in the placebo arm, *n* = 30. Normalized iMg concentration, which is adjusted for blood pH. Solid lines (black) are the mean difference ± 3 standard deviations; the long dash line (gray) is the fitted values; the short dash line (black) is the reference line for the mean difference of 0 mmol/L.

Baseline concentrations of iMg and tMg were correlated at r = 0.50 (*p*-value < 0.001). Using linear regression, the slope between iMg (outcome) and tMg (predictor) was 0.417 (intercept = 0.187); the slope was 0.422 (intercept = 0.186) when adjusted for treatment group and age stratum. Figure 1 provides a scatterplot of iMg and tMg measurements at baseline, which shows a positive and even scatter across the association between baseline iMg and tMg. Bland-Altman plots show the comparative agreement between the change in iMg and tMg in response to supplementation stratified by the treatment group (Figure 2). In the treatment group, there were slight variations between the changes in iMg across the mean of iMg measurements. Specifically, those with lower averaged iMg measurements tended to have positive change in iMg. There were not clear patterns for iMg in the placebo group or change in tMg.

### 3.4. Comparisons of Ionized Magnesium in Fresh, Refrigerated, and Frozen Blood Specimens

There were 39 participants with baseline iMg measured in fresh whole blood specimens as well as in stored samples. The average time from the blood draw to the analysis of baseline samples was 71 ± 29 min for refrigerated serum while for frozen serum it was 84 ± 15 days. Overall, the average iMg concentration was 0.54 ± 0.05 mmol/L in fresh whole blood samples; 0.68 ± 0.04 when measured in refrigerated samples; and 0.73 ± 0.05 in frozen serum samples, respectively. The mean pH was higher in refrigerated samples (7.45) and frozen samples at (7.53) compared to baseline whole blood samples (7.38).

**Figure 3:**
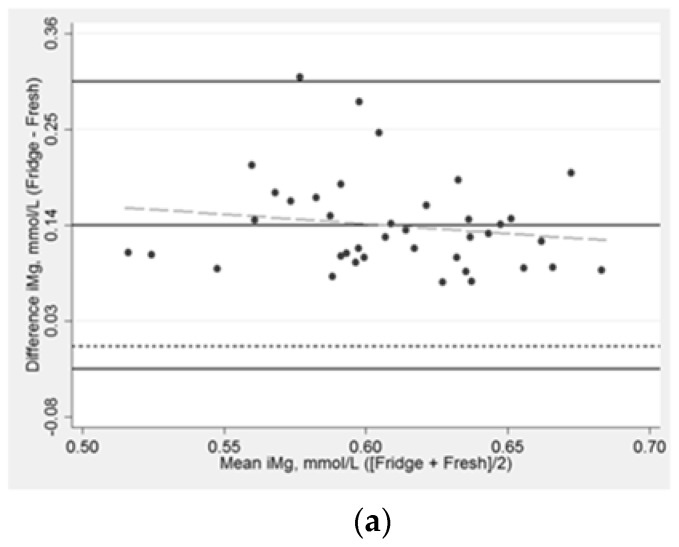

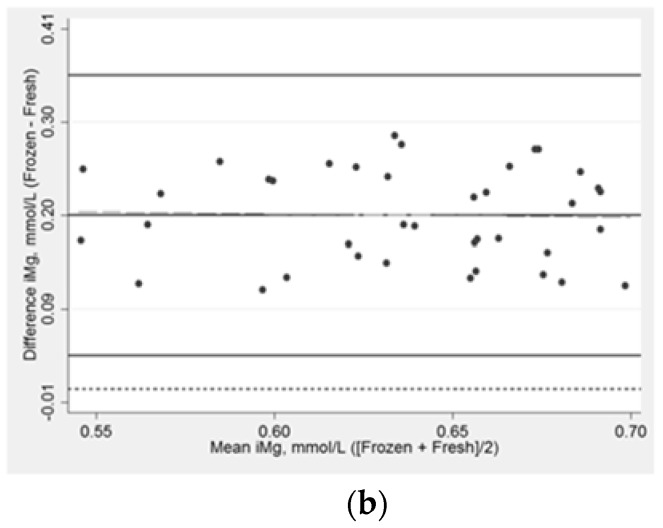
Bland-Altman plot assessing the (**a**) association between ionized magnesium concentrations at baseline in fresh whole blood and in serum after refrigeration, *n* = 39; (**b**) the association between ionized magnesium concentrations at baseline in fresh whole blood and in serum following one freeze–thaw cycle, *n* = 39. Normalized iMg concentration, which is adjusted for blood pH. The median time in the fridge = 71 min. The median time frozen = 81 days. Solid lines (black) are the mean difference ± 3 standard deviations; the long dash line (gray) is the fitted values; the short dash line (black) is the reference line for the mean difference of 0 mmol/L.

After refrigeration, serum iMg concentrations were higher compared to the iMg measured in fresh whole blood (mean: 0.14 mmol/L; 95% CI:0.12, 0.16). Following one freeze–thaw cycle, serum iMg was higher than in fresh whole blood by an average of 0.20 mmol/L (95% CI:0.18, 0.21). Bland-Altman plots depict the comparative agreement between iMg in whole blood analyzed within 10 min after blood draw, iMg in serum refrigerated for approximately 1 h (Figure 3a), and iMg after one freeze–thaw cycle (Figure 3b). The difference in iMg measured in refrigerated vs. fresh was higher by about 0.14 mmol/L and did not appreciably differ by the average of the two measurements (Table 3), while previously frozen vs. fresh was consistently higher by about 0.20 mmol/L. The difference between iMg using refrigerated vs. fresh samples ranged from 0.07 mmol/L to 0.30 mmol/L, while for frozen vs. fresh differences ranged from 0.11 to 0.29 mmol/L. Pearson’s partial correlations were r = 0.34 (*p*-value = 0.04) for refrigerated versus fresh samples and r = 0.46 (*p*-value = 0.005) for frozen versus fresh samples.

## 4. Discussion

In this randomized controlled trial, oral Mg supplementation over 10 weeks increased iMg in whole blood compared to placebo. The change did not differ by baseline iMg concentration, though we were not powered to detect subgroup differences. As previously reported [11], Mg supplementation results in increased tMg. With regard to specimen stability, concentrations of iMg measured in fresh whole blood were consistently overestimated based on refrigerated and frozen sera samples. 

The distribution of iMg in this relatively healthy population is largely consistent with those from other studies. One study suggested a reference interval for whole blood iMg of 0.44–0.59 mmol/L among 125 healthy participants, while among 200 consecutively recruited intensive care unit patients, the range of iMg concentrations was wider (0.35–0.78 mmol/L) [14]. Another study measured iMg in plasma using the same assay in the previous study [14] and reported a higher range of 0.53–0.67 mmol/L [15]. However, currently, there is not an established threshold for defining optimal iMg, particularly in relation to predicting longer-term health outcomes. Whether iMg is the more clinically relevant biomarker of ‘true’ Mg status also needs to be clarified, as it is possible that there may be substantial misclassification with tMg, resulting in attenuated effect estimates of evaluations of the association between Mg and cardiovascular disease and the misidentification and treatment of hypomagnesemia in clinical settings.

Four small oral Mg supplement RCTs (range *N* randomized = 26–60), conducted primarily in populations with comorbidities, have included blood measurements of both iMg and tMg [7,8,9,10]. Some of these studies utilized different iMg assays, which complicates the ability to draw comparisons between RCTs incorporating iMg. In a RCT of 60 elderly participants with type 2 diabetes [7], those randomized to 1 month of Mg supplementation experienced a statistically significant increase in iMg (but not tMg) from baseline, relative to placebo [7]. Other RCTs have found no effect of Mg supplementation on the biodistribution of circulating tMg or iMg [8,9,10]. However, in addition to being small, the studies are heterogeneous in terms of Mg dose/formulation, population, duration, and the specimen used for the iMg analysis (plasma, serum); thus, the RCTs incorporating both iMg and tMg are difficult to compare directly [7,8,9,10].

Most observational studies that have examined iMg in populations with medical conditions (e.g., chronic kidney disease, hypertension, pre-term labor) have done so cross-sectionally [4]. Many of these conditions are known to influence tMg concentrations [4]. A pilot study among 173 surgical intensive care unit patients reported poor agreement (weighted kappa = 0.35) between iMg and tMg status (low, normal, or high) [16]. It warrants mention, importantly, that the classifications of low, normal, or high tMg were based on established clinical cut-points, while the classifications of iMg as low, normal, or high were based on a reference interval derived from a healthy population [14].

In the present manuscript, we also evaluated the impact of specimen stability and processing on iMg concentrations. When iMg was analyzed using refrigerated and frozen serum, iMg concentrations were higher when compared to the whole blood iMg measured immediately after blood draw (as recommended by the assay manufacturer), and the pattern of higher iMg in refrigerated and frozen serum did not vary across iMg concentrations. If iMg was a more appropriate biomarker of ‘true’ Mg status, then plausibly iMg could be measured using stored serum and then corrected by a processing method, such as storage in the refrigerator or freezer. It is equally important to compare similarities or differences in circulating iMg by specimen type (i.e., whole blood, serum, plasma). Previously, in a study published in 1996, using an earlier generation of this iMg assay, the laboratory stability of iMg was analyzed in a cross-sectional analysis among relatively healthy participants under a variety of conditions (uncapped at room temperature, capped at room temperature, and capped at 4 °C) after 2, 4, and 6 h storage [14]. The average fresh whole blood iMg (0.52 mmol/L) was similar when measured after storage in capped tubes for 2–6 h at room temperature or at 4 °C. Compared to the iMg measured in a fresh blood specimen, mean iMg concentrations were similar when capped at room temperature and 4 °C; however, mean iMg was lower in uncapped room temperature samples. The pH of the blood increased over time (similar to our specimen stability results), particularly in uncapped samples, possibly due to CO_2_ loss [14]. In that study, iMg was not corrected to a pH of 7.4 [14]. Discrepancies in our findings could possibly relate to calibration or instrument differences. Further research is needed to determine if iMg is a stronger predictor of health outcomes, and, if so, whether this strength outweighs the challenges of using stored samples. The specialized equipment and higher cost of measuring iMg also needs to be considered.

It is important to be cognizant of the limitations of this analysis. Firstly, given how little research has been conducted on iMg, optimal concentrations of iMg, specifically in relation to health outcomes, are not well characterized. Secondly, the sample size in the present analysis is modest. We were powered to detect the overall effects of Mg supplementation but not subgroup comparisons, such as differences according to baseline iMg status (above/below median). Lastly, fresh iMg (i.e., iMg measured immediately after blood draw) was measured in whole blood, while serum was used for the measurements of iMg following storage in refrigerated and frozen conditions. It is possible that differences in specimen may account for the apparent difference in fresh iMg versus iMg in stored serum. However, previously, one study noted minimal differences in fresh iMg measured in whole blood (mean = 0.60 mmol/L), plasma (0.59 mmol/L), or serum (0.58 mmol/L) [17]. Nevertheless, the major strengths of this analysis are that it is one of the first randomized controlled supplement trials to examine both tMg and iMg in a relatively healthy population. Additionally, we were able to examine the (lack of) specimen stability of iMg when using refrigerated or frozen samples in a RCT.

## 5. Conclusions

In conclusion, we found that Mg supplementation over a 10-week period resulted in increased iMg concentrations. The baseline concentrations of iMg and tMg were modestly and positively associated. Using refrigerated and frozen serum, iMg concentrations consistently overestimated iMg compared to the measurements made in fresh whole blood. Whether iMg is a more appropriate measure of Mg status than tMg, and the public health and the clinical utility of measuring iMg remains to be determined. Further research is needed to learn how (or if) iMg relates to longer-term health outcomes and whether iMg is a better predictor of health outcomes than tMg.

## Figures and Tables

**Table 1:** Baseline participant characteristics stratified by study arm and by baseline ionized magnesium concentration (above vs. below the median), *n* = 59.

	Intervention Status		Baseline iMg Concentration	
	Magnesium (400 mg Daily)	Placebo	≥Median ^1^	<Median
*N*	29	30	28	26
Age, years ^2^	61.3 ± 5.3	61.6 ± 5.2	61.0 ± 4.3	62.2 ± 6.0
Age category				
≥65 years	6 (20.7)	8 (26.7)	5 (17.9)	8 (30.8)
<65 years	23 (79.3)	22 (73.3)	23 (82.1)	18 (69.2)
Sex				
Female	25 (86.2)	18 (60.0)	23 (82.1)	16 (61.5)
Male	4 (13.8)	12 (40.0)	5 (17.9)	10 (38.5)
Race				
White	27 (93.1)	29 (96.7)	26 (92.9)	25 (96.2)
Non-white	2 (6.9)	1(3.3)	2 (7.1)	1(3.8)
Education				
High school graduate or GED	0 (0.0)	1 (3.3)	0 (0.0)	0 (0.0)
Some college	6 (20.7)	4 (13.3)	4 (14.3)	5 (19.2)
College graduate	10 (34.5)	16 (53.3)	10 (35.7)	14 (53.9)
Graduate or professional school	13 (44.8)	9 (30.0)	13 (46.4)	7 (26.9)
BMI, kg/m^2^	27.7 ± 4.9	28.0 ± 4.5	26.9 ± 3.2	28.4 ± 5.4
Systolic blood pressure, mmHg	118.4 ± 14.9	119.3 ± 18.4	116.8 ± 12.1	122.0 ± 20.4
Diastolic blood pressure, mmHg	71.9 ± 8.7	71.2 ± 10.2	71.0 ± 7.0	72.46 ± 11.2
Glucose, mg/dL	94.2 ± 10.6	103.2 ± 40.2	94.1 ± 9.4	104.9 ± 43.1
Sensitivity analysis ^3^	94.2 ± 10.6	96.2 ± 11.64	94.1 ± 9.4	96.8 ± 12.7
pH	7.38 ± 0.02	7.38 ± 0.03	7.38 ± 0.02	7.38 ± 0.03
Total magnesium, mmol/L	0.86 ± 0.06	0.85 ± 0.05	0.87 ± 0.05	0.84 ± 0.06
Ionized magnesium, mmol/L ^4^	0.56 ± 0.06	0.55 ± 0.04	0.59 ± 0.03	0.51 ± 0.04
Total calcium, mmol/L	2.35 ± 0.09	2.34 ± 0.09	2.34 ± 0.09	2.35 ± 0.08
Ionized calcium, mmol/L ^4^	1.19 ± 0.03	1.18 ± 0.03	1.19 ± 0.03	1.18 ± 0.03

^1^ iMg median = 0.55 mmol/L; ^2^ N (%) or mean ± standard deviation; ^3^ omission of one participant with a baseline glucose value of 307 mg/dL; ^4^ ionized calcium and magnesium are both ‘normalized’ to pH 7.4. Abbreviations: GED, general education diploma; BMI, body mass index; iMg, ionized magnesium.

**Table 2:** Ten-week change in ionized and total magnesium concentrations by treatment group.

	Intervention Status		Mean Intervention Effect (95% CI) ^1^	*p*-Value
	Magnesium (400 mg Daily) Mean (SD)	Placebo Mean (SD)		
*N*	29	30		
iMg, mmol/L ^2^	22	27	0.03 (0.01, 0.05)	0.009
Baseline	0.56 (0.06)	0.54 (0.04)		
Follow-up ^3^	0.57 (0.03)	0.53 (0.04)		
Change	0.01 (0.05)	−0.01 (0.05)		
tMg, mmol/L	24	30	0.04 (0.01, 0.06)	0.004
Baseline	0.86 (0.06)	0.85 (0.05)		
Follow-up ^3^	0.89 (0.06)	0.85 (0.05)		
Change	0.03 (0.05)	0.00 (0.05)		

^1^ Adjusted for age (≥65 or <65) and baseline concentration (e.g., when the change in iMg was the outcome, models were adjusted for baseline iMg). The numbers of the observations included in the linear models are 49 and 54 for the outcomes ionized magnesium (whole blood) and total magnesium (serum); ^2^ normalized iMg concentration, which is adjusted for blood pH; ^3^ follow-up information obtained at intervention week 10. Abbreviations: SD, standard deviation; CI, confidence interval; iMg, ionized magnesium; tMg, total magnesium.

**Table 3:** Mean ionized magnesium concentrations in fresh, refrigerated and frozen blood samples and mean difference from baseline (fresh) after refrigeration and freezing, *n* = 39.

	Time from Draw to Analysis ^1^	pH ^1^	Concentrations, ^1^ mmol/L	Mean Difference (95% CI), mmol/L	Percentiles of Difference from Fresh iMg, mmol/L ^2^				
					Min	25th	50th	75th	Max
**iMg (normalized)**									
Fresh ^3^	4.8 min (3.4)	7.38 (0.03)	0.54 (0.05)	Reference	-	-	-	-	-
Refrigerated	69.6 min (25.3)	7.45 (0.04)	0.68 (0.04)	0.14 (0.12,0.16)	0.07	0.10	0.13	0.17	0.31
Frozen	82.2 days (15.4)	7.51 (0.04)	0.73 (0.05)	0.19 (0.18,0.21)	0.11	0.15	0.19	0.25	0.29
**iMg (not normalized)**									
Fresh	4.8 min (3.4)	7.38 (0.03)	0.54 (0.05)	Reference	-	-	-	-	-
Refrigerated	69.6 min (25.3)	7.45 (0.04)	0.65 (0.04)	0.11 (0.10,0.13)	0.04	0.07	0.09	0.13	0.25
Frozen	82.2 days (15.4)	7.51 (0.04)	0.68 (0.05)	0.14 (0.12,0.15)	0.05	0.09	0.13	0.18	0.20

^1^ Mean (standard deviation); ^2^ the distribution (in percentiles) of the difference between refrigerated vs. fresh and frozen vs. fresh. A value of 0 indicates that the iMg measured in refrigerated (or frozen) and fresh were identical. ^3^ Refrigerated and frozen blood samples were measured in serum, while fresh was measured in whole blood.

## Supplementary Materials

The following are available online at https://www.mdpi.com/2072-6643/12/1/263/s1, Figure S1: title, Table S1: Ten-week change in ionized and total magnesium concentrations by treatment group and stratified by baseline magnesium concentrations, Table S2 Ten-week change in ionized and total magnesium concentrations by treatment group, overall and stratified by baseline magnesium concentrations, excluding those who did not take > 80% capsules as assigned, *n* = 38.
