# Supplementary material for: Circulating Ionized Magnesium: Comparisons with Circulating Total Magnesium and the Response to Magnesium Supplementation in a Randomized Controlled Trial
Source: Nutrients. 2020 Jan 20;12(1):263. doi: 10.3390/nu12010263 (PMC7019442; doi:10.3390/nu12010263)

Supplemental Table 1. Ten-week change in ionized and total magnesium concentrations by treatment group and stratified by baseline magnesium concentrations

	Baseline Mg concentration ≥ median [a]				Baseline Mg concentration < median [a]				
	Intervention status				Intervention status				
	Magnesium (400 mg daily) Mean (SD)	Placebo Mean (SD)	Mean Intervention Effect (95% CI)[b]	p-value	Magnesium (400 mg daily) Mean (SD)	Placebo Mean (SD)	Mean Intervention Effect (95% CI)	p-value	p-interaction
iMg,[c] mmol/L	14	13	0.03 (0.00, 0.07)	0.07	8	14	0.03 (0.00, 0.07)	0.07	0.86
Baseline	0.59 (0.03)	0.57 (0.01)			0.50 (0.04)	0.51 (0.03)			
Follow-up[d]	0.58 (0.02)	0.54 (0.05)			0.55 (0.04)	0.53 (0.04)			
Change	-0.01 (0.02)	-0.03 (0.05)			0.06 (0.04)	0.01 (0.04)			
tMg, mmol/L	16	17	0.05 (0.01, 0.08)	0.01	8	13	0.02 (-0.00, 0.05)	0.08	0.27
Baseline	0.89 (0.04)	0.88 (0.02)			0.79 (0.03)	0.80 (0.03)			
Follow-up[d]	0.92 (0.05)	0.87 (0.05)			0.83 (0.04)	0.82 (0.05)			
Change	0.03 (0.05)	-0.02 (0.05)			0.05 (0.03)	0.02 (0.04)			

Abbreviations: SD, standard deviation; CI, confidence interval; iMg, ionized magnesium; tMg, total magnesium; [a] iMg median = 0.55 mmol/L; tMg median = 0.86 mmol/L; [b] Adjusted for age (≥65 or <65), and baseline concentration (e.g. when change in iMg is the outcome, models were adjusted for baseline iMg). The numbers of observations included in linear models for baseline Mg ≥ median are 27 and 33 for the outcomes iMg (whole blood) and tMg (serum), for baseline Mg < median, the number of observations was 22 and 21 for the outcomes iMg and tMg; [c] Normalized iMg concentration which is adjusted for blood pH; [d] Follow-up information obtained at intervention week 10.

Supplemental Table 2. Ten-week change in ionized and total magnesium concentrations by treatment group, overall and stratified by baseline magnesium concentrations, excluding those who did not take >80% capsules as assigned, n=38

	**Treatment arm**				**Baseline Mg concentrations [a]**				
					**≥ median**		**< median**		
	Magnesium (400 mg daily) Mean (SD)	Placebo Mean (SD)	Mean Intervention Effect (95% CI)[b]	p-value	Mean Intervention Effect (95% CI)	p-value	Mean Intervention Effect (95% CI)	p-value	p-interaction
N	15	23							
iMg,[c] mmol/L	11	22	0.04 (0.01, 0.06)	0.007	0.04 (-0.01, 0.09)	0.14	0.06 (0.01, 0.10)	0.02	0.37
Baseline	0.57 (0.06)	0.54 (0.04)							
Follow-up[d]	0.58 (0.02)	0.54 (0.04)							
Change	0.01 (0.05)	-0.01 (0.05)							
tMg, mmol/L	13	23	0.05 (0.02, 0.08)	0.003	0.05 (0.01, 0.10)	0.02	0.03 (-0.00, 0.07)	0.07	0.47
Baseline	0.85 (0.05)	0.85 (0.05)							
Follow-up[d]	0.89 (0.06)	0.84 (0.06)							
Change	0.04 (0.04)	-0.01 (0.05)							

Abbreviations: SD, standard deviation; CI, confidence interval; iMg, ionized magnesium; tMg, total magnesium; [a] iMg median = 0.55 mmol/L; tMg median = 0.86 mmol/L; [b] Adjusted for age (≥65 or <65), and baseline concentration (e.g. when change in iMg is the outcome, models were adjusted for baseline iMg). The numbers of observations included in linear models are 49 and 54 for the outcomes ionized magnesium (whole blood) and total magnesium (serum); [c] Normalized iMg concentration which is adjusted for blood pH; [d] Follow-up information obtained at intervention week 10.

Supplementary Materials

Supplemental Figure 1. Study participant flow chart

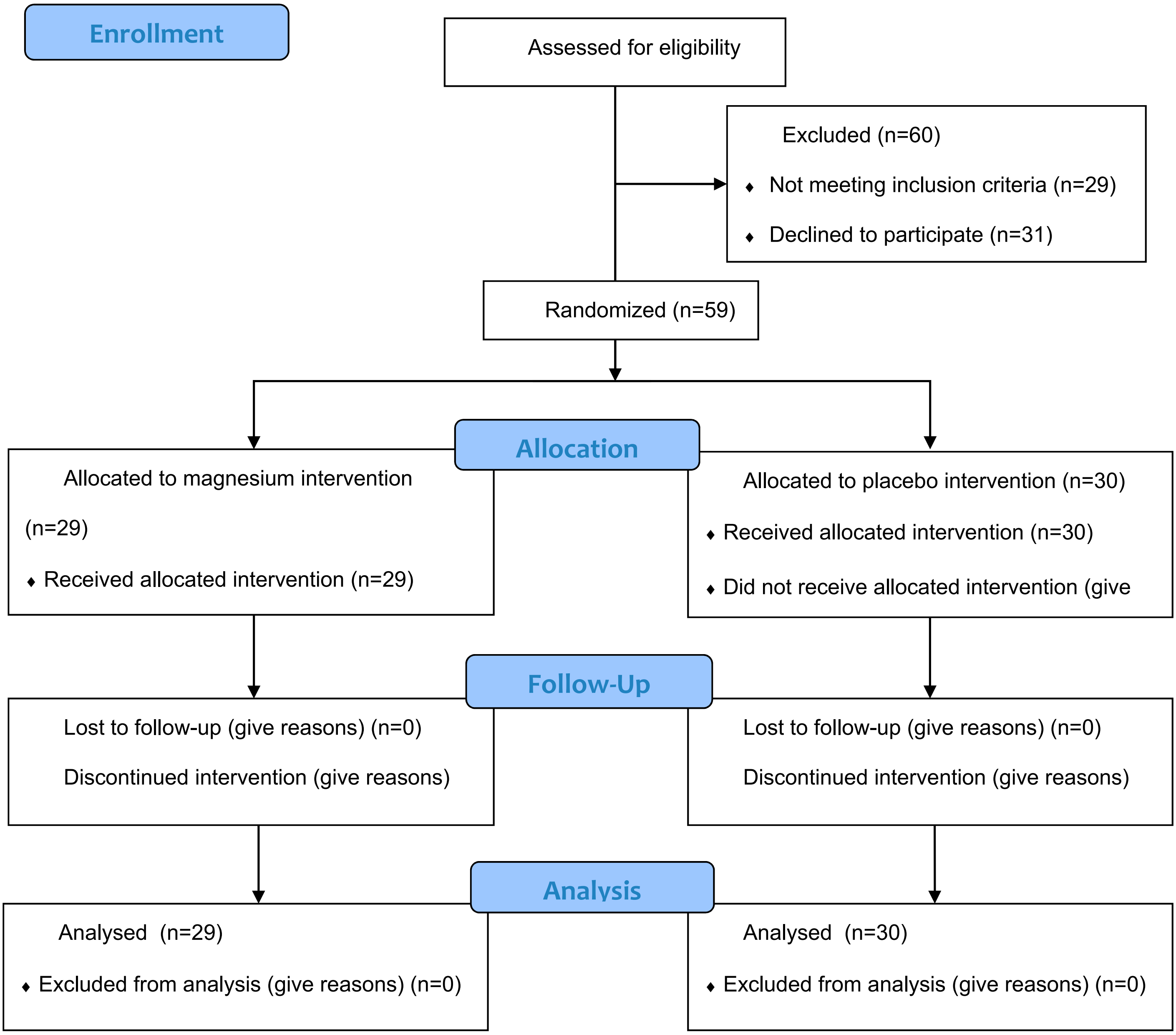

Supplement: Supplementary file 1 [file nutrients-12-00263-s001.pdf]
